# Radiocarbon chronology and environmental context of Last Glacial Maximum human occupation in Switzerland

**DOI:** 10.1038/s41598-020-61448-7

**Published:** 2020-03-13

**Authors:** Hazel Reade, Jennifer A. Tripp, Sophy Charlton, Sonja Grimm, Kerry L. Sayle, Alex Fensome, Thomas F. G. Higham, Ian Barnes, Rhiannon E. Stevens

**Affiliations:** 1 UCL Institute of Archaeology, 31-34 Gordon Square, London, WC1H 0PY United Kingdom; 2 Department of Earth Sciences, Natural History Museum, Cromwell Road, London, SW7 5BD United Kingdom; 3 Centre for Baltic and Scandinavian Archaeology, Stiftung Schleswig-Holsteinische Landesmuseen, Schloss Gottorf, D-24837 Schleswig, Germany; 4 Scottish Universities Environmental Research Centre, Rankine Avenue, East Kilbride, G75 0QF United Kingdom; 5 Research Laboratory for Archaeology and the History of Art, University of Oxford, Dyson Perrins Building, South Parks Road, Oxford, OX1 3QY United Kingdom

**Keywords:** Environmental sciences, Archaeology, Climate sciences, Palaeoclimate

## Abstract

Central Europe during the Last Glacial Maximum (LGM) was dominated by polar desert and steppe-tundra biomes. Despite this, a human presence during this time period is evident at several locations across the region, including in Switzerland, less than 50 km from the Alpine ice sheet margin. It has been hypothesised that such human activity may have been restricted to brief periods of climatic warming within the LGM, but chronological information from many of these sites are currently too poorly resolved to corroborate this. Here we present a revised chronology of LGM human occupation in Switzerland. AMS radiocarbon dating of cut-marked reindeer (*Rangifer tarandus*) bones from the sites of Kastelhöhle-Nord and Y-Höhle indicates human occupation of Switzerland was most likely restricted to between 23,400 and 22,800 cal. BP. This timeframe corresponds to Greenland Interstadial 2, a brief warming phase, supporting the hypothesis that human presence was facilitated by favourable climatic episodes. Carbon, nitrogen and sulphur stable isotope analysis of the fauna provides palaeoenvironmental information for this time period. These findings contribute to our understanding of human activity in ice-marginal environments and have implications for understanding cultural connections across central Europe during the LGM.

## Introduction

**Figure 1:**
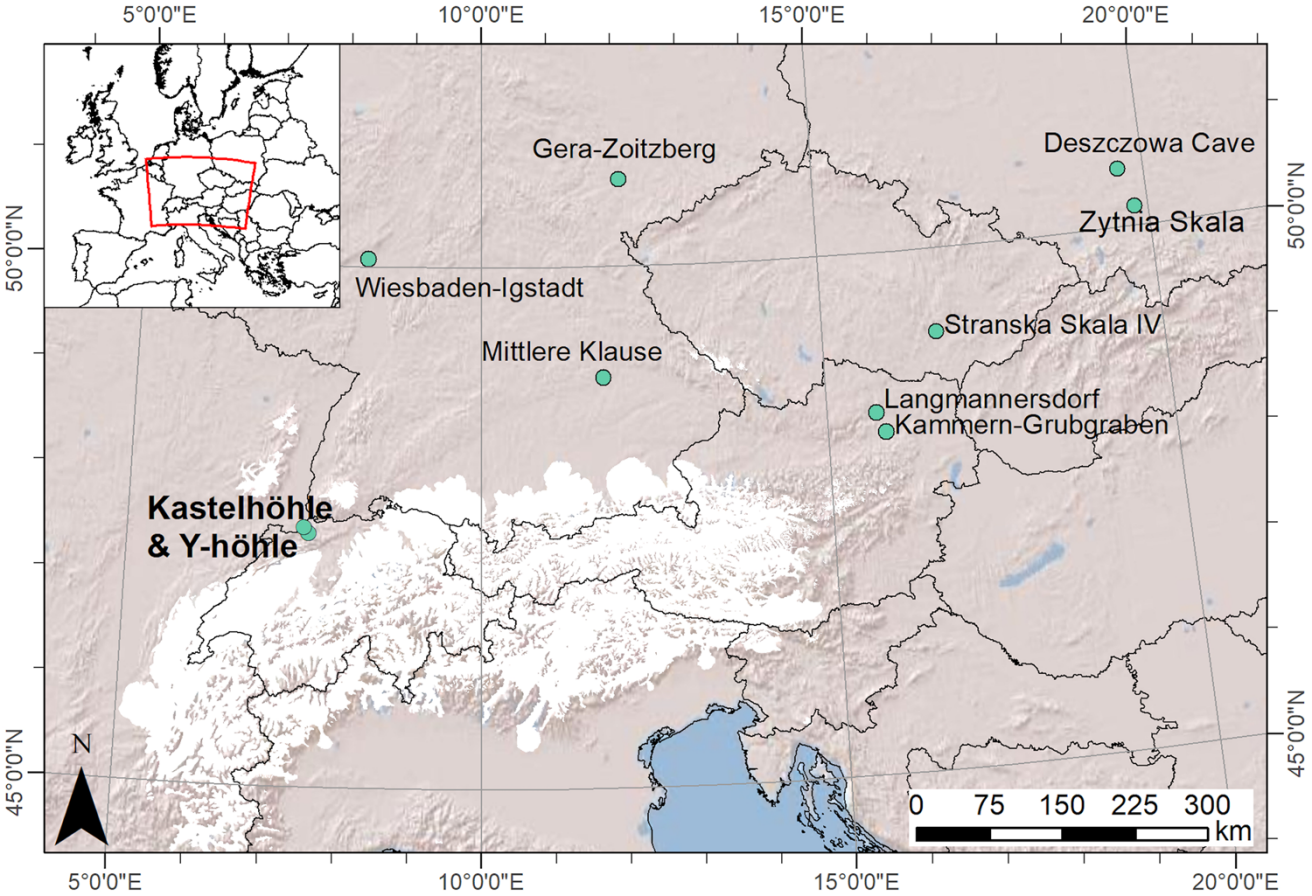
Location of Kastelhöhle-Nord and Y-Höhle shown in relation to the other archaeological localities discussed in the text. Last Glacial Maximum Alpine ice sheet extent is indicated in white^81^. Inset: location of study area in Europe indicated by red box. Map created in ArcMap 10.5 (https://desktop.arcgis.com/en/arcmap/). Shaded Relief basemap from Esri (https://services.arcgisonline.com/ArcGIS/rest/services/World_Shaded_Relief/MapServer).

Switzerland during the Last Glacial Maximum (LGM, c. 26,500–19,000 BP^1,2^) was almost entirely covered by ice, with only a small region north of the Jura Mountains remaining ice-free^3^. Despite this, human occupation during this period is evidenced from two locations in Switzerland; Kastelhöhle-Nord and Y-Höhle^4,5^ (Fig. 1). Previous radiocarbon dates from these sites indicate that people were present between 24,000 and 22,000 cal. BP^5,6^. This broadly corresponds to a period of global glacial advance, when a cold-arid climate existed in Europe north of the Alps, and mean annual temperatures were as much as 15 °C lower than present-day^7,8^. However, short-term climatic fluctuations are also evident during this time interval, including a brief warming event associated with Greenland Interstadial 2 (GI-2, c. 23,300–22,800 BP^9^), which may have facilitated human occupation in Switzerland^6^. If this hypothesis is to be tested, more precise chronological and environmental information regarding the human activity at Kastelhöhle-Nord and Y-Höhle is required. This information is also crucial to situate the Swiss LGM archaeological record within the wider debate surrounding the extent of human occupation in central Europe during the LGM, the environmental conditions under which it occurred, and the extent to which different cultural groups may have interacted^6^. Here we present a revised chronology of human occupation in Switzerland during the LGM based on new radiocarbon dates, and environmental interpretations based on stable isotope analysis (carbon, nitrogen and sulphur) of reindeer bone collagen.

The LGM is a time-transgressive period within Marine Isotope Stage 2 (MIS2). The nomenclature is variously used to refer to the episode during which maximum global ice volume was reached, when minimum global sea level occurred, or when the lowest oxygen isotope values (δ^18^O) are recorded in marine or ice core records^10,11^. Associated time intervals range from c. 25,000 to 18,000 BP, but broadly centre upon 21,000 BP^10,11^. Within Switzerland, the last glacial advanced occurred sometime after 30,000 BP^1^. Local geomorphological evidence and speleothem δ^18^O minima suggest maximum ice extent may have been reached between 26,500 and 22,000 BP, while the onset of rapid ice decay started by c. 19,000 BP^1,2,12,13^. Climate models and proxy data indicate a cold dry climate existed north of the Alps during this time period, while the formation of Alpine speleothems suggests warm air masses from the south delivered precipitation to the northern Alpine slopes^2,7,8^. North of the mountains, continuous periglacial permafrost conditions existed, with tundra and steppe landscapes dominated by graminoid and forb vegetation^14,15^. Nonetheless, the Alpine LGM was punctuated by sub-millennial climate fluctuations related to changing temperature and precipitation regimes, which influenced local environmental conditions and ice extent^2,16–18^. Within Switzerland a positive excursion in speleothem δ^18^O occurred between 23,230 ± 37 BP and 22,759 ± 47 BP^2^, linked to changing precipitation patterns, and this appears to have coincided with a phase of recession in the Alpine ice sheet^18^. The timings of these local environmental changes matches with the Greenland warm period of GI-2 (23,290 ± 596 BP to 22,850 ± 573 BP^9^), within dating uncertainties. This suggests that the warming in Switzerland may have been associated with continental-scale processes. The Greenland record of GI-2 is interrupted by a brief climatic cooling lasting around 200 years (Greenland Stadial 2.2) and centring on approximately 23,100 BP^9^. The expression of this event in the Swiss record is less clear but may occur between 22,971 ± 46 BP and 22,888 ± 50 BP^2^.

The link between GI-2 and human activity in Switzerland has previously been postulated^6^. However, chronological resolution is currently poor and archaeological evidence is sparse. The dated cut-marked reindeer (*Rangifer tarandus*) bones from Kastelhöhle-Nord (n = 3) and Y-Höhle (n = 1) represent the only directly dateable archaeological evidence from this time period in Switzerland^5,6^. Both cave sites are located within 10 km of one another, on the north-eastern edge of the Jura Mountains, less than 50 km from the maximum ice sheet extent. Lithics found in association with the reindeer bones at Kastelhöhle-Nord have been attributed to the Badegoulian/Early Magdalenian^4,6^, a Late Upper Palaeolithic culture found in western-central Europe. In contrast, the cut-marked reindeer bone from Y-Höhle was found in a secondary context with no association to other archaeological remains^5^. Lithics of Badegoulian character are also found in Germany (Wiesbaden-Igstadt, Rhineland, and Gera-Zoitzberg, Thuringia)^6,19^, while broadly contemporaneous evidence of human activity is also present in southern Germany (Mittlere Klause), and further east in Austria (Kammern-Grubgraben and Langmannersdorf), the Czech Republic (Stranska Skala IV) and possibly Poland (Deszczowa Cave and Zytnia Skala)^20–23^. The latter are usually attributed to Epigravettian cultures of eastern central Europe^21^. Hence, a great amount of uncertainty surrounds the cultural and chronological relationship between these sites, as well as the environmental conditions under which they were occupied, but it has been postulated that brief climatic warming events may have facilitated the periodic expansions of human populations into northern central Europe, and cultural contact across it^6^.

## Radiocarbon and stable isotope analyses

One of the most significant developments in the radiocarbon dating of bone collagen in the last 20 years has been the routine inclusion of an ultrafiltration step in the sample pre-treatment procedure at some laboratories^24,25^. Ultrafilters remove the low molecular weight fraction from the sample, thus more thoroughly removing modern organic contaminants^24^. However, not all studies conclude that ultrafiltration is a necessary part of the radiocarbon bone sample preparation procedure^26,27^, and without stringent cleaning protocols their use may risk introducing additional contaminants into the sample^24,28,29^. Certainly, it is necessary to employ rigorous quality checks to the radiocarbon dating process, regardless of the method of sample preparation used^30,31^. Following such quality controls, the application of the ultrafiltration method has been shown to have particularly significant implications for the dating of bone collagen from Palaeolithic contexts, where the contribution of even the smallest amount of contamination can have significant consequence for the obtained date^32–34^. Considering these developments, it is timely to re-evaluate the radiocarbon chronology of human occupation in Switzerland during the LGM. As such, the four previously dated cut-marked reindeer bones from Kastelhöhle-Nord intermediate horizon and Y-Höhle were targeted for radiocarbon dating using the current ultrafiltration sample preparation methodology and quality control criteria used by the Oxford Radiocarbon Accelerator Unit (ORAU).

Bone collagen can also be analysed for its stable isotope compositions, which are powerful tools for investigating past ecology and terrestrial environments, and have been employed widely in Late Pleistocene archaeological research^35–41^. In this study we use carbon (δ^13^C), nitrogen (δ^15^N) and sulphur (δ^34^S) isotope ratios in reindeer bone collagen to examine paleoenvironmental conditions during the period of human activity at Kastelhöhle-Nord and Y-Höhle. Carbon isotope ratios are largely determined by dietary behaviour, and reindeer δ^13^C values are known to be systematically enriched in comparison to other herbivore species due the consumption of lichen^42,43^. Dietary δ^13^C values are also influenced by atmospheric CO_2_ δ^13^C value and concentration, and by environmental parameters such as temperature, moisture availability and density of vegetation cover^35,44–46^. Bone collagen δ^15^N values are linked to both dietary specialisation^41,47,48^ and to environmental conditions^29–32^. Soil and plant δ^15^N values are influenced by climatic variables such as temperature and precipitation, mediated through soil processes^49,50^. In particular, soil maturity, nutrient availability and microbial activity have been cited as having a strong control on herbivore bone collagen δ^15^N values, with permafrost and proximity to ice sheets likely playing a significant role in the generally low δ^15^N values observed in Late Pleistocene Europe^36–39^. Bone collagen δ^34^S values largely reflect the varying forms of sulphur present in soil, which is related to mineral input from the underlying geology, deposition of sulphates from groundwater and the atmosphere, and microbially-mediated fractionation processes^51^. As such, δ^34^S values are spatially variable and often considered a tool for exploring mobility and landscape utilisation^38,52,53^. However, bone collagen δ^34^S values may also hold significant promise as a palaeoenvironmental proxy representing changing hydrological and microbial processes^51,54,55^. In this study, bone collagen stable isotope analyses are performed on the 4 dated specimens, plus a further 7 reindeer bones from Kastelhöhle-Nord intermediate horizon.

## Results

Collagen preservation at both sites was excellent; all samples produced collagen yields between 2.9% and 14.3%, which is comparable to the amount of collagen produced from modern samples prepared using the same methodology^56^. All samples had C:N atomic ratios between 3.2 and 3.4, and carbon and nitrogen content between 41–45% and 14–16%, respectively, comparable to *in vivo* collagen^57,58^. Sulphur content ranged between 0.14–0.19%, while C:S and N:S atomic ratios were between 625–785 and 190–245, indicating good sample integrity^59^.

**Figure 2:**
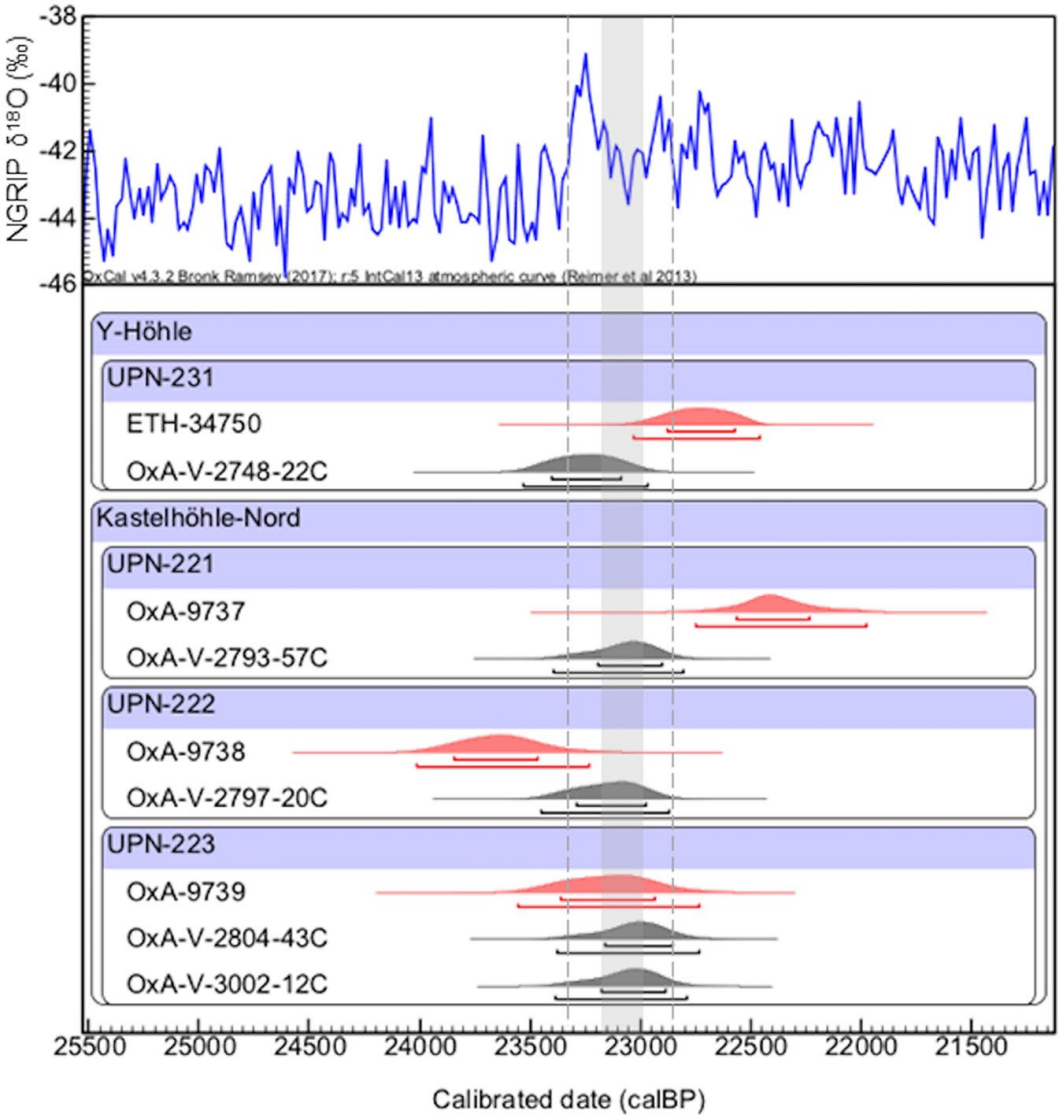
Calibrated radiocarbon dates made on cut-marked reindeer bones from Kastelhöhle-Nord and Y-Höhle (Table 1). Calibration performed using OxCal 4.3^82^ and the INTCAL13^83^ dataset and shown against the NGRIP δ^18^O record^84,85^. Dates in red are previously published radiocarbon dates^5,6^, dates in grey are new radiocarbon dates from this study. Dashed lines indicate duration of Greenland Interstadial 2, which is intermediated by Greenland Stadial 2.2 (grey shading). The OxA-V-xxxx-xxC codes denote dates on collagen extracted at UCL, corrected for measured modern background carbon (full details are given in supplementary information 3).

**Figure 3:**
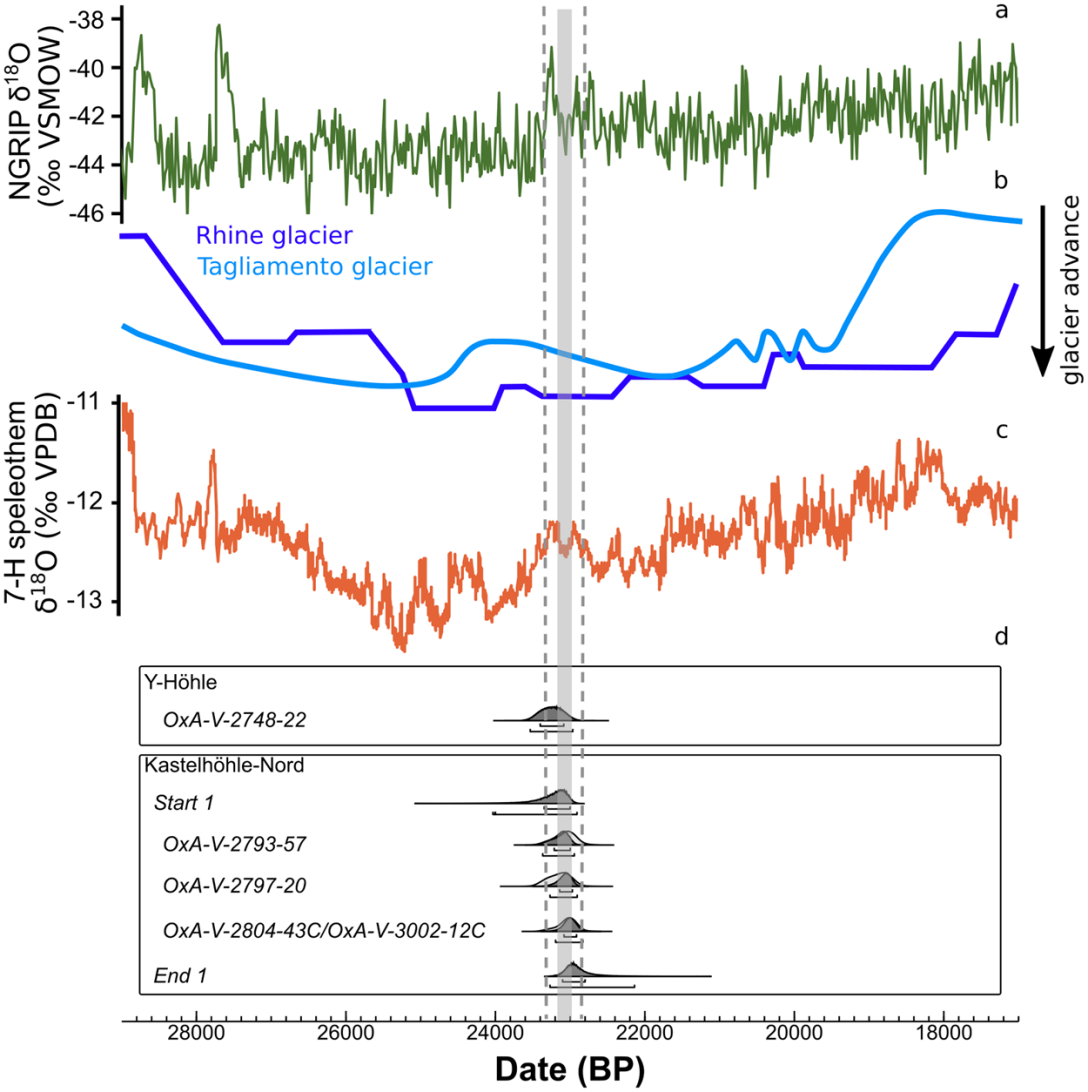
(**a**) NGRIP δ^18^O record;^84,85^. (**b**) Alpine glacier advances reconstructed for Tagliamento and Rhine glaciers;^1,18^. (**c**) combined Alpine speleothem 7H-2 and 7H-3 δ^18^O record;^2^ (**d**) Bayesian single-phase model for Kastelhöhle-Nord, shown alongside the calibrated date for Y-Höhle. Data modelled using OxCal 4.3^82^ and the INTCAL13^83^ dataset. Dashed lines indicate duration of Greenland Interstadial 2, which is intermediated by Greenland Stadial 2.2 (grey shading). The OxA-V-xxxx-xxC codes denote dates on collagen extracted at UCL, corrected for measured modern background carbon (full details are given in supplementary information 3).

The new radiocarbon determinations fall within the range of the previously published dates, but indicate a significantly shorter duration of human activity (Table 1, Fig. 2). Two new radiocarbon dates were produced on sample UPN-223 as an internal laboratory quality check. As the results are statistically identical an error-weighted mean of 19,121 ± 60 ^14^C BP shall be used for this sample in the subsequent discussion of the results. The overall range represented by the new radiocarbon dates is 19,300 ± 90 ^14^C BP to 19,121 ± 60 ^14^C BP (Table 1). A Bayesian statistical modelling approach was applied to the Kastelhöhle-Nord samples assuming that the three samples represent the same phase of activity at the site. The results show that occupation occurred between 23,450 and 22,733 cal. BP (95% probability). This compares to the date from Y-Höhle of 23,531–22,963 cal. BP (95% probability) (Fig. 3).

**Table 1:** AMS radiocarbon determinations and isotope ratio mass spectrometer (IRMS) δ^13^C isotope measurements obtained during the dating procedure, made on bone collagen from the cut-marked reindeer remains found at Kastelhöhle-Nord and Y-Höhle.

Project Sample Code	Species	Element	Collagen yield (%)	δ^13^C	C/N	AMS Code	^14^C years BP	Calibrated Age BP (95% probability)	Reference
**Y-Höhle**									
UPN-231	*Rangifer tarandus*	metatarsal, cut-marked	not given	−23.5*	not measured	ETH-34750	18,875 ± 115	23,028–22,462	^5^
			2.9	−19.9	3.3	OxA-V-2748-22C	19,300 ± 90	23,531–22,963	this study
**Kastelhöhle-Nord**									
UPN-221	*Rangifer tarandus*	metatarsal, cut-marked	8.2	−18.5	3.1	OxA-9737	18,530 ± 150	22,746–21,975	^6^
			8.8	−19.0	3.3	OxA-V-2793-57C	19,140 ± 80	23,392–22,806	this study
UPN-222	*Rangifer tarandus*	tibia, cut-marked	7.4	−18.3	3.5	OxA-9738	19,620 ± 140	24,016–23,230	^6^
			10.6	−18.9	3.3	OxA-V-2797-20C	19,200 ± 90	23,450–22,867	this study
UPN-223	*Rangifer tarandus*	phalanx 1, cut-marked	8.2	−18.4	3.4	OxA-9739	19,200 ± 150	23,550–22,730	^6^
			12.9	−19.1	3.3	OxA-V-2804-43C	19,110 ± 90	23,379–22,733	this study
			12.9	−19.1	3.3	OxA-V-3002-12C	19,130 ± 80	23,382–22,790	this study

*δ^13^C for ETH-34750 (UPN-231) was measured using AMS not IRMS.

**Figure 4:**
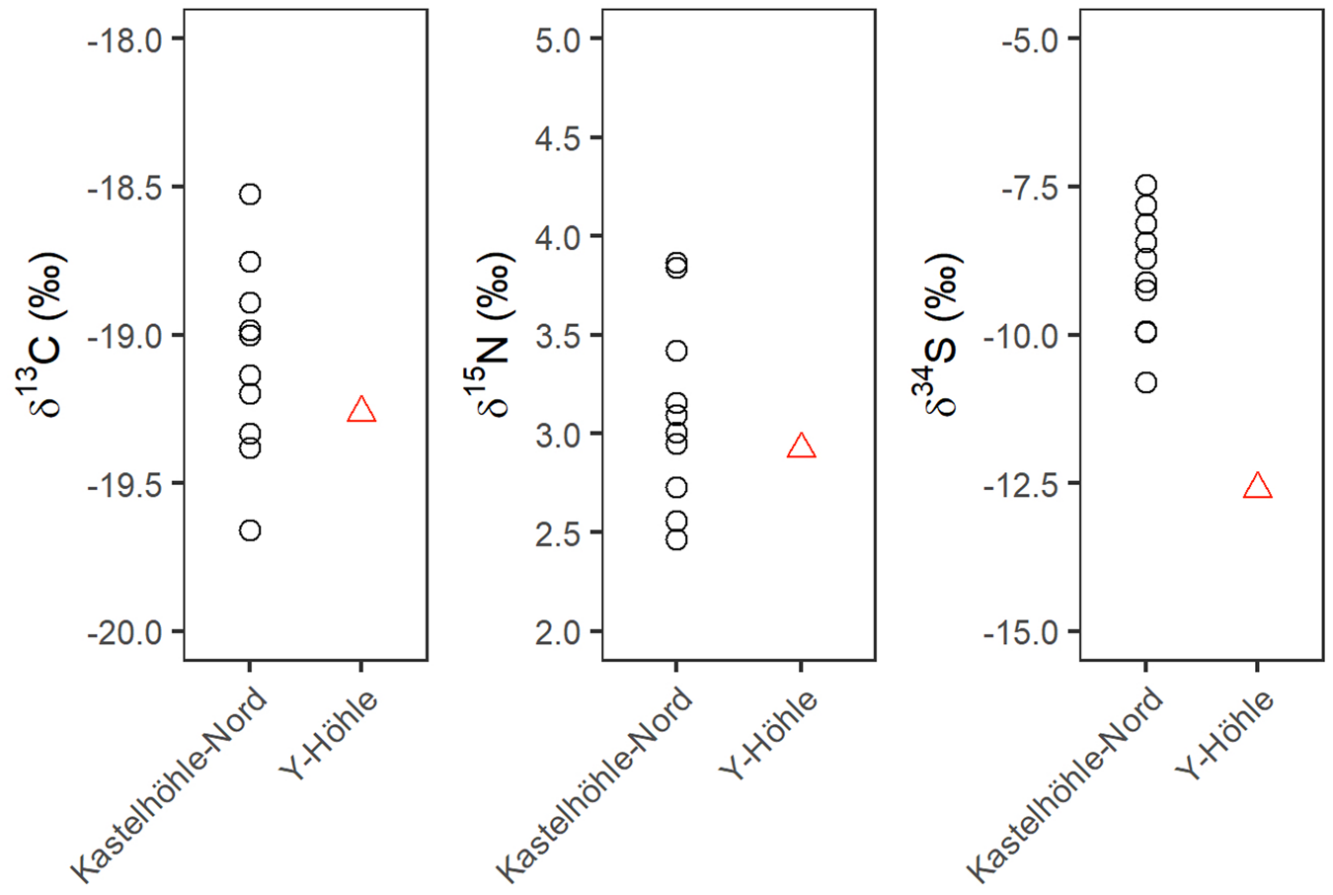
Carbon (δ^13^C), nitrogen (δ^15^N) and sulphur (δ^34^S) stable isotope results from the analysed reindeer bones from Kastelhöhle-Nord and Y-Höhle.

For stable isotope analyses, each sample was analysed in duplicate and reproducibility was better than ±0.1‰ for δ^13^C, ±0.2‰ for δ^15^N and ±0.3‰ for δ^34^S. Reindeer δ^13^C and δ^15^N values from the Kastelhöhle-Nord intermediate horizon and Y-Höhle ranged from –19.7‰ to –18.5‰ and +2.5‰ to +3.9‰ respectively (Fig. 4, Supplementary Table S1). These values are typical of Late Pleistocene reindeer in Europe^36–38^ and largely reflect the animals’ behavioural ecology and dietary specialisation for lichen, underlain by environmental influences. Reindeer δ^34^S values from the Kastelhöhle-Nord intermediate horizon ranged from –10.8‰ to –7.5‰, while the δ^34^S value for the Y-Höhle sample was measured as –12.6‰ (Fig. 4, Supplementary Table S1).

## Discussion

### Chronology of human settlement in Switzerland and central Europe during the LGM

Based on the new radiocarbon evidence, human settlement in Switzerland took place between c. 23,400 and 22,800 cal. BP. While the possibility of human presence in Switzerland during the LGM and early post-LGM outside of this time interval cannot be excluded, currently no evidence exists to support this. The radiocarbon age range broadly coincides with the end of a phase of Alpine glacier recession (c. 23,500 BP^1,18^), and with a period of change to Alpine precipitation patterns (23,230 ± 37 BP to 22,759 ± 47 BP^2^). This phase of Alpine environmental change likely corresponds to Greenland Interstadial 2 (23,290 ± 596 BP to 22,850 ± 573 BP^9^), although the dating uncertainties do no permit correlation with absolute certainty. Nonetheless, the age range of the faunal radiocarbon dates does appear to correlate well with the local, short-lived episode of climatic amelioration and glacial recession (Fig. 3), suggesting human presence in Switzerland at this time may have been facilitated by environmental change. However, even the smaller uncertainties associated with these local records means that we cannot be certain that the phase of human activity corresponds to the interstadial. This is where the isotope data derived directly from the cut-marked specimens can facilitate our interpretation of local environmental conditions at the time of human activity.

The slightly older date from Y-Höhle compared to Kastelhöhle-Nord (and perhaps rather speculatively, the different δ^34^S values between the sites), indicates that human activity during this time period was likely not restricted to a single occupation event. Indeed, while the Y-Höhle sample was found in a secondary context with no association to other archaeological remains^5^, the Kastelhöhle-Nord samples were found in association with a lithic assemblage of 228 artefact^5^, which could represent multiple periods activity at the site. The impact of the cooler phase that occurred within GI-2 (GS-2.2, dated locally to c. 22,971 ± 46 BP to 22,888 ± 50 BP) cannot be assessed given the short duration of the event relative to the dating uncertainties.

Although sparse, the archaeological record of central Europe after c. 25,000 BP does attest to human activity persisting during the LGM part of MIS 2, at least sporadically^6,21^. Similarities between the Swiss lithic record and those from Germany (Wiesbaden-Igstadt and Gera-Zoitzberg) and lower Austria (Kammern-Grubgraben) have been interpreted as indicating long-distance contact across western and eastern central Europe during this time, and this is supported by the overlapping radiocarbon chronologies of the sites (with the exception of Gera-Zoitzberg where there is no absolute chronological information available)^6,19^. More broadly, radiocarbon dating of archaeological material from Mittlere Klause in southern Germany, Langmannersdorf in Austria and Stranska Skala IV in the Czech Republic also span this time interval^6,20,22^.

Recent chronological investigations at Kammern-Grubgraben (Layers 2–4) have produced a series of 6 ultrafiltered AMS dates ranging from 19,330 ± 70 ^14^C BP to 19,070 ± 60 ^14^C BP (c. 23,400 and 22,850 cal. BP), significantly refining the likely duration of human activity from a previous estimate of 19,380 ± 90 ^14^C BP to 17,350 ± 190 ^14^C BP^23^. This revised chronology shows striking overlap with the Swiss dates, and with GI-2. By comparison, Wiesbaden-Igstadt, Mittlere Klause, Langmannersdorf and Stranska Skala IV all have radiocarbon chronologies that span this time interval, but with age ranges too broad to evaluate whether a more certain relationship with GI-2 can be established. Indeed, the only ultra-filtered date from Mittlere Klause (OxA-9856 18590 ± 260 ^14^C BP^4^) was later withdrawn due to concerns over potential contamination^60^. It would certainly be of interest to re-date this sample and revisit the chronology of other sites to further evaluate the timing of human activity in central Europe during the LGM.

### Environmental context of human settlement in Switzerland during the LGM

The δ^13^C values obtained from the Swiss reindeer samples are characteristic of a diet incorporating a significant proportion of lichen^37,38^. Lichens are typically enriched in ^13^C by c. 2–4‰, relative to C_3_ vascular plants growing within the same environment^61^, and this enrichment is reflected in reindeer skeletal δ^13^C, relative to other herbivores with a diet based solely on C_3_ vascular plants^42,43^. For many modern reindeer herds lichen is an important food source, particularly in the winter months when reindeer forage for the resource under snow cover^62^. It is likely that the ability to utilise lichen as a significant dietary component facilitated the survival of reindeer close to the ice sheet margins. Their use of this habitat could also explain the reason for human presence in the region during the LGM. However, it is not possible to evaluate whether reindeer, or indeed people, remained year-round in the vicinity of Kastelhöhle-Nord and Y-Höhle. Both sedentary and long-distance seasonal migratory behaviours are observed in modern reindeer herds^63–65^ and a similar diversity of behaviours has been argued for European Pleistocene populations^66–69^. It is therefore possible that human presence in northern Switzerland represents only short-term, perhaps seasonally-restricted, exploitation of local reindeer herds during periods of favourable environmental conditions.

Regardless of whether the reindeer represent a sedentary or migratory population, their δ^15^N and δ^34^S values provide information on the environment in which they lived. In the context of the Jura Mountains during the Late Pleistocene, soil maturity has been suggested as the controlling factor on reindeer δ^15^N isotope ratios, with low values (≤2‰) indicating recently deglaciated, nutrient limited landscapes and higher values (≥3.5‰) indicating reindeer were utilising refugial areas, where soils were more developed^38^. The majority of the LGM Kastelhöhle-Nord and Y-Höhle reindeer display intermediary δ^15^N values (+2.5‰ to +3.9‰). We suggest that the observed δ^15^N values could be produced in an environment where climatic/permafrost conditions limited soil nutrient cycling, which would have produced low environmental δ^15^N values, but where landscape stability facilitated the development/survival of soils and hardy vegetation enough to sustain reindeer populations. There is a smaller amount of variation in the δ^34^S values for the Swiss reindeer than in other LGM populations^38,70^, suggesting the Swiss animals had a more homogenous geographical range. However, it is interesting to note that of the four directly dated samples, the oldest (from Y-Höhle) has the lowest δ^34^S value (–12.6‰), while the three slightly younger Kastelhöhle-Nord samples have an average δ^34^S value of –8.2 ± 0.4‰, potentially indicating a temporal change in local environmental conditions or migratory behaviour.

**Figure 5:**
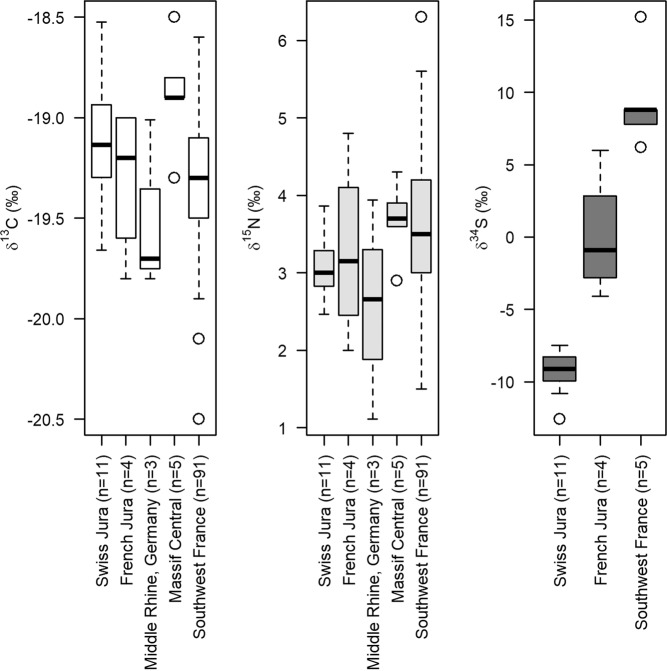
Carbon (δ^13^C), nitrogen (δ^15^N) and sulphur (δ^34^S) stable isotope results from the analysed reindeer bones from this study (Swiss Jura), compared to previously published data from the French Jura^38^, Middle Rhine in Germany^36^, Massif Central in France^70^ and Southwest France^70,86–88^.

When results are compared to published reindeer collagen stable isotope data from other regions in Europe during the LGM, considerable overlap in δ^13^C and δ^15^N values between locations is observed, suggesting reindeer ecology and behaviour plays a significant role in the derived isotopic signatures (Fig. 5, Supplementary Table S2). The highest δ^13^C values are observed in reindeer from the Swiss and French Jura and the Massif Central in France, which may indicate a greater reliance on lichen as a food source in these environments, or potentially a small altitudinal effect on the carbon isotope signatures^71^. δ^15^N values overlap between all regions considered, but the lowest (≤2‰) and highest (≥5‰) δ^15^N values that are observed in the Middle Rhine region of Germany and southwest France respectively, are absent from the Swiss and French Jura and the Massif Central samples. While the lack of the highest δ^15^N values suggests an environment in which soil nutrient cycling was limited, probably due to overall low environmental temperatures, the lack of the lowest δ^15^N values also suggests periglacial processes likely did not dominate the environmental δ^15^N signature^36,38^. Significant location-based differences are apparent in the δ^34^S values (Fig. 5, Supplementary Table S2), although the comparatively small sample size used in the data comparison should be noted (n = 9 for δ^34^S, compared to n = 103 for δ^13^C and δ^15^N). Reindeer δ^34^S values reflect the soil δ^34^S values upon which they fed. Soil δ^34^S values are related to underlying lithology and can be altered through changing rates of mineral weather and soil-bedrock interactions, controlled by hydrological conditions and bacterially-mediated reduction and oxidation processes^51,54,55^. The surface lithology of the regions compared (southwest France, northern Swiss Jura, and French Jura) are underlain by similar late Mesozoic and Cenozoic limestone and sandstone deposits^72^, which are unlikely to vary in δ^34^S values to the extent observed in the reindeer data. This suggests environmental parameters are influential in the observed signal. Low environmental δ^34^S values have been related to low oxygen/water-logged soil conditions, though the processes that govern these fractionations are complex and not yet fully understood^55,73–75^. It is therefore possible that the lower δ^34^S values observed in the Swiss samples, compared to the French samples, could indicate an environment that had experienced wetter/more oxygen limited soil conditions. Such conditions could have been brought about by changing Alpine precipitation regimes, as identified in local speleothem records^2^, or by localised permafrost thaw related to the (albeit small) increased temperatures of GI-2.

## Conclusion

The refined chronological model presented here, based on current available archaeological evidence, indicates that the LGM human occupation of Switzerland was likely restricted to a brief period of local climatic warming, most probably associated with Greenland Interstadial 2. This supports the hypothesis put forward by Terberger and Street^6^. Stable isotope analysis of reindeer bone collagen, directly associated with human activity, further supports this interpretation. While the data indicates the landscapes in which humans hunted reindeer remained comparatively cold, it also suggests relatively stable environmental conditions. Compared to other European reindeer data, the higher carbon isotope values suggest a greater reliance on lichen by the Swiss reindeer, indicating that other vegetation types may have been more limited. The low sulphur and intermediary nitrogen isotope values could represent the influence of reduced/changing soil bacterial activity related to a brief respite in the most severe climatic conditions associated with the global LGM and local glacial advance. Combined, this data supports the hypothesis that human activity in Switzerland was made possible by a phase of climatic warming that occurred during the part of MIS2 that is more broadly characterised by global glacial advance.

While the chronologies of many of the central European LGM archaeological sites are currently too imprecise to permit robust comparison, the striking similarities between the revised chronology for Switzerland and that recently published for Kammern-Grubgraben in Austria^23^, offer a tantalising insight into what could be a relatively brief episode of LGM human activity across central Europe, facilitated by the warmer environmental conditions that GI-2 provided. Whether the other known LGM archaeological sites in central Europe also correspond to this warmer period, or whether they represent different episodes of human activity during the LGM, can only be further examined through better chronological characterisation of these sites. Additionally, we stress the importance of also obtaining location-specific palaeoenvironmental records when making inferences about the types of landscapes in which human occupation took place.

## Methods

0.2 to 1.3 g of bone sample was collected from each specimen using a dental drill. Collagen extraction was performed at University College London (UCL) using a modified version of the Oxford Radiocarbon Accelerator Unit (ORAU) collagen extraction procedure (AF)^31^, which is based on a modified version of the Longin^75^ method. All samples were treated with 0.5 M hydrochloric acid (HCl) at 4 °C until fully demineralised (24hrs – 2 weeks), then thoroughly rinsed with ultrapure water. Demineralised samples were then gelatinised in pH3 HCl solution at 75 °C for 48hrs and filtered using a pre-cleaned Ezee-filter. The filtrate was passed through a pre-cleaned 15–30 kD ultrafilter, with the >30 kD fraction collected and lyophilised.

For stable isotope analysis, 1.2–1.5 mg aliquots of purified collagen were weighed into tin capsules and analysed using a Delta V Advantage continuous-flow isotope ratio mass spectrometer coupled via a ConfloIV to an EA IsoLink elemental analyser (Thermo Fisher Scientific, Bremen) at the Scottish Universities Environmental Research Centre (SUERC). For every ten unknown samples, three in-house standards that are calibrated to the International Atomic Energy Agency (IAEA) reference materials USGS40 (L-glutamic acid, δ^13^C_VPDB_ = –26.4‰, δ^15^N_AIR_ = –4.5‰), USGS41 (L-glutamic acid, δ^13^C_VPDB_ = +37.6‰, δ^15^N_AIR_ = –47.6‰), USGS43 (Indian Human Hair: δ^15^N_AIR_ = +8.44‰, δ^13^C_VPDB_ = –21.28‰, δ^34^S_VCDT_ = +10.46‰), IAEA-S-2 (silver sulfide, δ^34^S_VCTD_ = +22.7‰), and IAEA-S-3 (silver sulfide, δ^34^S_VCTD_ = –32.3‰) were analysed^76^. Results are reported as per mil (‰) relative to the internationally accepted standards VPDB, AIR and VCDT. Measurement uncertainty was determined to be ±0.1‰ for δ^13^C, ±0.2‰ for δ^15^N, and ±0.3‰ for δ^34^S on the basis of repeated measurements of an in-house bone collagen standard and a certified fish gelatin standard (Elemental Microanalysis, UK).

Radiocarbon dating was performed at ORAU using their standard procedures, as described by Brock *et al*.^31^. Approximately 5 mg of purified collagen was weighed into a tin capsule that had been baked at 500 °C for 12 hours. Samples were combusted using an elemental analyser coupled to an isotope ratio mass spectrometer, employing a splitter to allow for collection of the CO_2_ ^31,77^. Samples were graphitised by reduction of collected CO_2_ over an iron catalyst in an excess H_2_ atmosphere at 560 °C^78,79^. ^14^C dates were measured on the Oxford AMS system using a caesium ion source for ionisation of the solid graphite sample^24^. To denote the bone pre-treatment at UCL rather than at ORAU, all measured dates were given “OxA-V-wwww-pp” numbers, where “wwww” indicates the wheel number, and “pp” is the position of the sample on the wheel^24^. As bone pre-treatment was performed in the laboratory at UCL dates were corrected for potential background contamination within this laboratory using two known-age reference samples^80^. Corrected dates are denoted by adding a “C” to the end of the date code assigned by ORAU. Uncorrected measured date values as well as further details of the correction calculations are provided in Supplemental Information 3. Details of the pre-treatment procedure used in the previous dating of these samples is given in Supplementary Information 4.

## Supplementary information

Supplementary information.

## Data Availability

All data is provided in the supplementary information.
