# Supplementary material for: Radiocarbon chronology and environmental context of Last Glacial Maximum human occupation in Switzerland
Source: Sci Rep. 2020 Mar 13;10:4694. doi: 10.1038/s41598-020-61448-7 (PMC7070022; doi:10.1038/s41598-020-61448-7)
Supplement: Supplementary file 1 — Supplementary information. [file 41598_2020_61448_MOESM1_ESM.pdf]

Radiocarbon chronology and environmental context of Last Glacial Maximum human occupation in Switzerland

Hazel Reade[1*], Jennifer A. Tripp[1], Sophy Charlton[2], Sonja Grimm[3], Kerry L. Sayle[4], Alex Fensome[1], Thomas F.G. Higham[5], Ian Barnes[2], Rhiannon E. Stevens[1]

[1] UCL Institute of Archaeology, 31-34 Gordon Square, London, WC1H 0PY, United Kingdom

[2] Department of Earth Sciences, Natural History Museum, Cromwell Road, London SW7 5BD, United Kingdom

[3] Centre for Baltic and Scandinavian Archaeology, Stiftung Schleswig-Holsteinische Landesmuseen, Schloss Gottorf, D-24837 Schleswig, Germany

[4] Scottish Universities Environmental Research Centre, Rankine Avenue, East Kilbride G75 0QF, United Kingdom

[5] Research Laboratory for Archaeology and the History of Art, University of Oxford, Dyson Perrins Building, South Parks Road, Oxford OX1 3QY, United Kingdom

[*]corresponding author: h.reade@ucl.ac.uk, +44(0)20 7679 1530

S1 Results from the stable isotope analyses performed on *Rangifer tarandus* bones in this study

Site	Sample code	Context Info.	Element	Collagen Yield (mg)	Collagen Yield %	%C	%N	%S	$\delta^{13}C$	$\delta^{15}N$	$\delta^{34}S$	C/N	C/S	N/S	Replicate analysis
Kastelhöhle	UPN-221	Intermediate horizon (Badegoulian)	metatarsus, cut-marked	54.9	8.8	43.8	15.6	0.19	-19.2	3.2	-8.7	3.3	628	192	2
Kastelhöhle	UPN-222	Intermediate horizon (Badegoulian)	tibia, cut-marked	84.4	10.6	43.3	15.5	0.18	-18.5	2.7	-7.8	3.2	659	203	2
Kastelhöhle	UPN-223	Intermediate horizon (Badegoulian)	phalange I, cut-marked	51.8	12.9	43.2	15.4	0.16	-18.8	2.6	-8.1	3.3	721	220	2
Kastelhöhle	UPN-224	Intermediate horizon (Badegoulian)	pelvis	63.1	12.5	44.5	15.4	0.17	-19.4	2.5	-7.5	3.4	715	213	2
Kastelhöhle	UPN-225	Intermediate horizon (Badegoulian)	tibia	74.5	13.0	43.1	15.2	0.17	-18.9	3.1	-9.9	3.3	678	205	2
Kastelhöhle	UPN-226	Intermediate horizon (Badegoulian)	long bone fragment	62.6	9.9	43.2	15.1	0.17	-19.3	3.8	-9.3	3.3	662	198	2
Kastelhöhle	UPN-227	Intermediate horizon (Badegoulian)	long bone fragment	60.0	9.2	42.9	15.3	0.16	-19.1	3.4	-10.0	3.3	700	213	2
Kastelhöhle	UPN-228	Intermediate horizon (Badegoulian)	long bone fragment	73.3	14.3	43.0	15.4	0.16	-19.0	3.0	-8.4	3.2	724	223	2
Kastelhöhle	UPN-229	Intermediate horizon (Badegoulian)	long bone fragment	59.2	11.4	42.8	14.8	0.18	-19.7	3.9	-10.8	3.4	649	193	2
Kastelhöhle	UPN-230	Intermediate horizon (Badegoulian)	long bone fragment	54.7	11.7	43.7	15.3	0.17	-19.0	2.9	-9.1	3.3	670	201	2
Y-höhle	UPN-231	59/20/1	metatarsal, cut-marked	20	2.9	41.8	15.3	0.14	-19.3	2.9	-12.6	3.2	781	245	2

S2 Stable isotope values used in the data comparison reported in Figure 5

Site	Region	Sample	δ13C	δ15N	δ34S	Reference
Abri Pataud	Southwest France	PAT-7	-19.1	4.9	8.8	70, 86
Abri Pataud	Southwest France	PAT-4	-19.3	4.7	7.8	70, 86
Abri Pataud	Southwest France	PAT-5	-19.2	3.8	15.2	70, 86
Abri Pataud	Southwest France	PAT-6	-19.5	4.4	8.8	70, 86
Abri Pataud	Southwest France	PAT-8	-18.7	4.3	6.2	70, 86
Baume Noire	French Jura	FRT-3	-19.0	4.8	6.0	38
Baume Noire	French Jura	FRT-2	-19.4	2.9	-1.5	38
Combe-Saunière	Southwest France	CS2600*	-19.1	4.1	no data	87
Combe-Saunière	Southwest France	CS2300*	-19.6	3.6	no data	87
Combe-Saunière	Southwest France	CS3100	-19.3	3.6	no data	87
Combe-Saunière	Southwest France	CS2800*	-19.3	3.0	no data	87
Combe-Saunière	Southwest France	CS2500*	-19.7	2.9	no data	87
Combe-Saunière	Southwest France	CS2700*	-19.2	2.9	no data	87
Combe-Saunière	Southwest France	CS2400*	-19.1	2.6	no data	87
Grotte Grappin/ Saint-Vincent's	French Jura	ARL 3	-19.0	2.0	-0.3	38
La Baume (Gigny-sur-Suran)	French Jura	GIS 2	-19.8	3.4	-4.1	38
Laugerie-Haute est	Southwest France	LGH900	-19.2	3.8	no data	87
Laugerie-Haute est	Southwest France	LGH1000	-18.6	3.7	no data	87
Laugerie-Haute est	Southwest France	LGHP2000	-19.0	3.4	no data	87
Laugerie-Haute est	Southwest France	LGH200	-19.0	3.3	no data	87
Laugerie-Haute est	Southwest France	LGH500	-19.6	3.2	no data	87
Laugerie-Haute est	Southwest France	LGHP1900	-19.3	3.1	no data	87
Laugerie-Haute est	Southwest France	LGHP1800	-18.9	3.0	no data	87
Laugerie-Haute est	Southwest France	LGH400	-19.7	2.8	no data	87
Laugerie-Haute est	Southwest France	LGH600	-19.3	2.8	no data	87
Laugerie-Haute est	Southwest France	LGHP2200	-19.4	2.8	no data	87
Laugerie-Haute est	Southwest France	LGHP2100	-19.4	2.6	no data	87
Laugerie-Haute est	Southwest France	LGH800	-19.1	2.0	no data	87
Laugerie-Haute est	Southwest France	LGH700	-19.5	1.5	no data	87
Laugerie-Haute est	Southwest France	LGH2800	-19.5	4.7	no data	87
Laugerie-Haute est	Southwest France	LGH1700	-19.5	4.6	no data	87
Laugerie-Haute est	Southwest France	LGH2900	-20.1	4.5	no data	87
Laugerie-Haute est	Southwest France	LGH2400	-19.4	4.3	no data	87
Laugerie-Haute est	Southwest France	LGH2200	-19.5	4.2	no data	87
Laugerie-Haute est	Southwest France	LGH3000	-19.5	4.1	no data	87
Laugerie-Haute est	Southwest France	LGH3100	-19.1	3.3	no data	87
Laugerie-Haute est	Southwest France	LGH300	-19.3	2.5	no data	87
Le Blot	Massif Central	BLO-29	-18.9	3.9	no data	70
Le Blot	Massif Central	BLO-30	-18.9	3.7	no data	70
Le Blot	Massif Central	BLO-31	-18.8	3.6	no data	70
Le Blot	Massif Central	BLO-27	-18.5	2.9	no data	70
Le Blot	Massif Central	BLO-14	-19.3	4.3	no data	70
Les Jamblancs	Southwest France	JBL1900	-19.4	3.6	no data	88
Les Jamblancs	Southwest France	JBL1500	-18.9	3.3	no data	88
Les Jamblancs	Southwest France	JBL400	-18.9	3.3	no data	88
Les Jamblancs	Southwest France	JBL1000	-18.6	3.2	no data	88
Les Jamblancs	Southwest France	JBL1400	-18.7	3.2	no data	88
Les Jamblancs	Southwest France	JBL900	-18.9	3.1	no data	88
Les Jamblancs	Southwest France	JBL1700	-19.4	3.0	no data	88
Les Jamblancs	Southwest France	JBL1800	-19.3	2.9	no data	88
Les Jamblancs	Southwest France	JBL1300	-19.3	2.7	no data	88
Les Jamblancs	Southwest France	JBL600	-19.1	2.7	no data	88
Les Jamblancs	Southwest France	JBL1100	-18.7	2.6	no data	88
Les Jamblancs	Southwest France	JBL3400	-19.5	5.2	no data	88
Les Jamblancs	Southwest France	JBL3100	-19.8	4.8	no data	88
Les Jamblancs	Southwest France	JBL3300	-19.6	3.6	no data	88
Les Jamblancs	Southwest France	JBL3500	-19.4	3.6	no data	88

Les Jamblancs	Southwest France	JBL3200	-19.7	2.2	no data	87
Les Peyrugues	Southwest France	PRG3900	-19.2	6.3	no data	87
Les Peyrugues	Southwest France	PRG2800	-19.1	4.6	no data	87
Les Peyrugues	Southwest France	PRG2900	-19.4	4.4	no data	87
Les Peyrugues	Southwest France	PRG1900	-19.2	4.0	no data	87
Les Peyrugues	Southwest France	PRG1100	-19.3	3.9	no data	87
Les Peyrugues	Southwest France	PRG2200	-19.3	3.9	no data	87
Les Peyrugues	Southwest France	PRG2600	-19.3	3.9	no data	87
Les Peyrugues	Southwest France	PRG1400	-19.4	3.7	no data	87
Les Peyrugues	Southwest France	PRG1800	-19.4	3.7	no data	87
Les Peyrugues	Southwest France	PRG1300	-19.7	3.6	no data	87
Les Peyrugues	Southwest France	PRG1600	-19.0	3.6	no data	87
Les Peyrugues	Southwest France	PRG1000	-19.2	3.5	no data	87
Les Peyrugues	Southwest France	PRG2300	-19.4	3.4	no data	87
Les Peyrugues	Southwest France	PRG2400	-19.3	3.3	no data	87
Les Peyrugues	Southwest France	PRG1200	-19.7	3.2	no data	87
Les Peyrugues	Southwest France	PRG1500	-19.4	3.2	no data	87
Les Peyrugues	Southwest France	PRG2100	-18.8	3.2	no data	87
Les Peyrugues	Southwest France	PRG2700	-20.5	3.2	no data	87
Les Peyrugues	Southwest France	PRG1700	-19.1	3.0	no data	87
Les Peyrugues	Southwest France	PRG2500	-19.8	2.9	no data	87
Les Peyrugues	Southwest France	PRG2000	-19.2	2.7	no data	87
Les Peyrugues	Southwest France	PRG4300	-19.5	5.6	no data	87
Les Peyrugues	Southwest France	PRG4400	-19.2	5.4	no data	87
Les Peyrugues	Southwest France	PRG3600	-18.8	4.9	no data	87
Les Peyrugues	Southwest France	PRG4500	-19.1	4.8	no data	87
Les Peyrugues	Southwest France	PRG4800	-19.0	4.8	no data	87
Les Peyrugues	Southwest France	PRG3400	-19.4	4.4	no data	87
Les Peyrugues	Southwest France	PRG3200	-19.3	4.3	no data	87
Les Peyrugues	Southwest France	PRG3300	-19.2	4.3	no data	87
Les Peyrugues	Southwest France	PRG3700	-19.3	4.3	no data	87
Les Peyrugues	Southwest France	PRG5000	-19.9	4.2	no data	87
Les Peyrugues	Southwest France	PRG4100	-19.6	4.0	no data	87
Les Peyrugues	Southwest France	PRG4200	-19.5	3.8	no data	87
Les Peyrugues	Southwest France	PRG4600	-18.7	3.8	no data	87
Les Peyrugues	Southwest France	PRG5200	-19.7	3.5	no data	87
Les Peyrugues	Southwest France	PRG3800	-19.4	3.4	no data	87
Les Peyrugues	Southwest France	PRG4000	-19.6	3.3	no data	87
Les Peyrugues	Southwest France	PRG4700	-19.2	3.3	no data	87
Les Peyrugues	Southwest France	PRG3100	-19.7	2.9	no data	87
Les Peyrugues	Southwest France	PRG5100	-19.7	2.7	no data	87
Les Peyrugues	Southwest France	PRG3500	-19.9	2.6	no data	87
Saint-Germain-la Rivière	Southwest France	SGR200	-19.2	3.5	no data	87
Wiesbaden-Igstadt	Middle Rhine, Germany	A/IG/B/1	-19.0	2.7	no data	36
Wiesbaden-Igstadt	Middle Rhine, Germany	OxA-7501	-19.8	3.9	no data	36
Wiesbaden-Igstadt	Middle Rhine, Germany	OxA-7500	-19.7	1.1	no data	36

S3 Radiocarbon corrections

Because the pretreatment of the radiocarbon samples was done at UCL, it was necessary to apply an additional correction to the dates produced at ORAU, to account for any contamination introduced into the samples during pretreatment. In general, we are concerned with detecting and correcting for two main sources of contamination: old or "radiocarbon dead" carbon contamination, which tends to come from solvents or oils in the lab with petroleum origin; and modern carbon that can come from a variety of sources.

With this in mind, two reference samples, one of a relatively recent known age, and one of an age beyond radiocarbon measurement, were routinely subject to parallel pretreatment at UCL along with our archaeological samples, and then analysed on the AMS at ORAU. Corrections were done according to the method of Wood, et al.[80].

Mary Rose cow rib bone

The error-weighted mean of ten radiocarbon measurements of collagen extracted from a cow rib recovered from the *Mary Rose* is 316 ± 7 BP. This is consistent with the calculated radiocarbon date of the ship sinking (311 BP), and the calibrated date in Figure S3.1 shows agreement with the historical date (1545 AD). The agreement of our measured *Mary Rose* dates with the accepted historical date indicates that our procedures did not introduce contamination with carbon from old, "radiocarbon dead", sources, and thus no corrections for old carbon were applied to our dates.

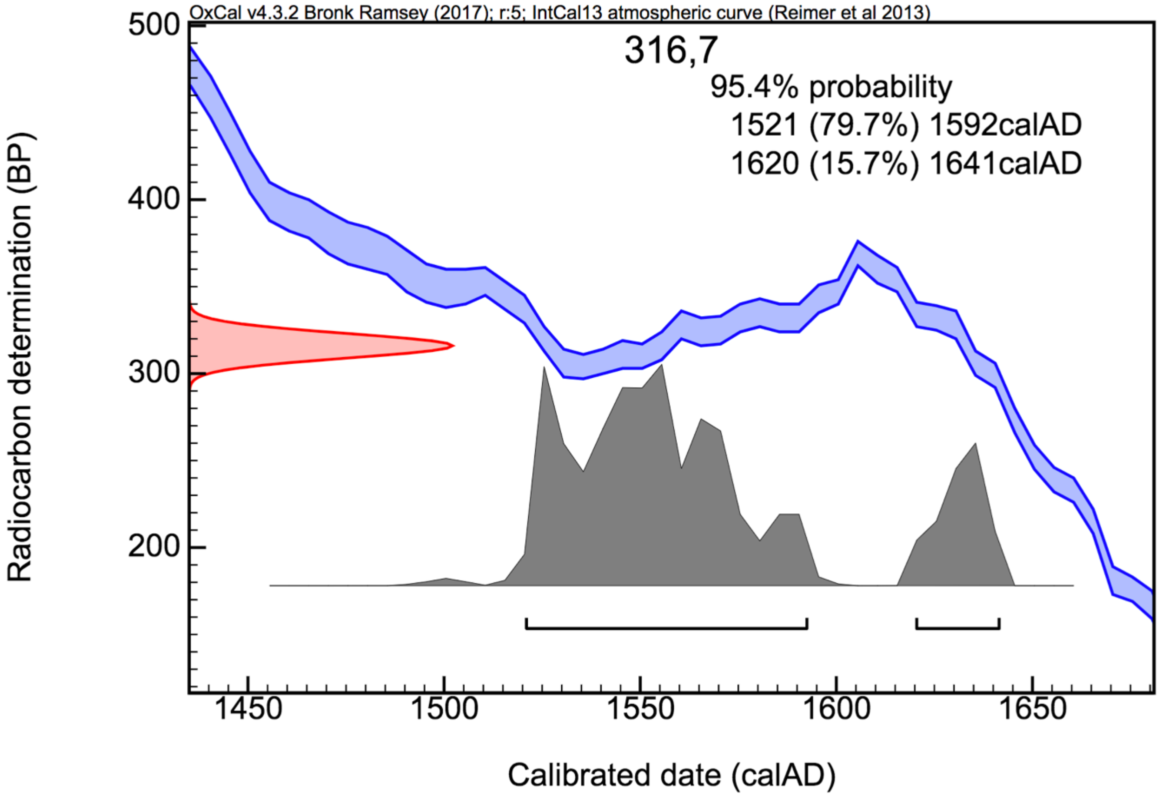

Figure S3.1 Probability range for the error-weighted mean date (316 ± 7 BP) on Mary Rose cow rib collagen. Calibration performed using OxCal 4.3.2[82]and the INTCAL13[83] dataset. Plot created in OxCal 4.3.2[82]

Latton Mammoth long bone (MIS7)

Radiocarbon measurements on fourteen collagen extractions from the Latton Mammoth long bone have been done. This bone dates from Marine Isotope Stage 7, and thus should not have any ^{14}C present. The error-weighted mean of the measured dates comes to 51,400 ± 750 BP, indicating a low but persistent background of modern contamination.

Figure S3.2 shows that the mg yield of collagen does not affect the amount of measured background carbon. Samples we date must have a yield of greater than 5 mg. In practice, all of the samples we have dated as part of this project have yields greater than 6.8 mg, with a mean value of 38.6 mg and median value of 30.1 mg. As seen in figure S3.2, the linear correlation between yield and percent modern carbon (pMC) is low (a very slight positive correlation with R^2=0.0149). We therefore applied the same background correction to each sample regardless of collagen yield.

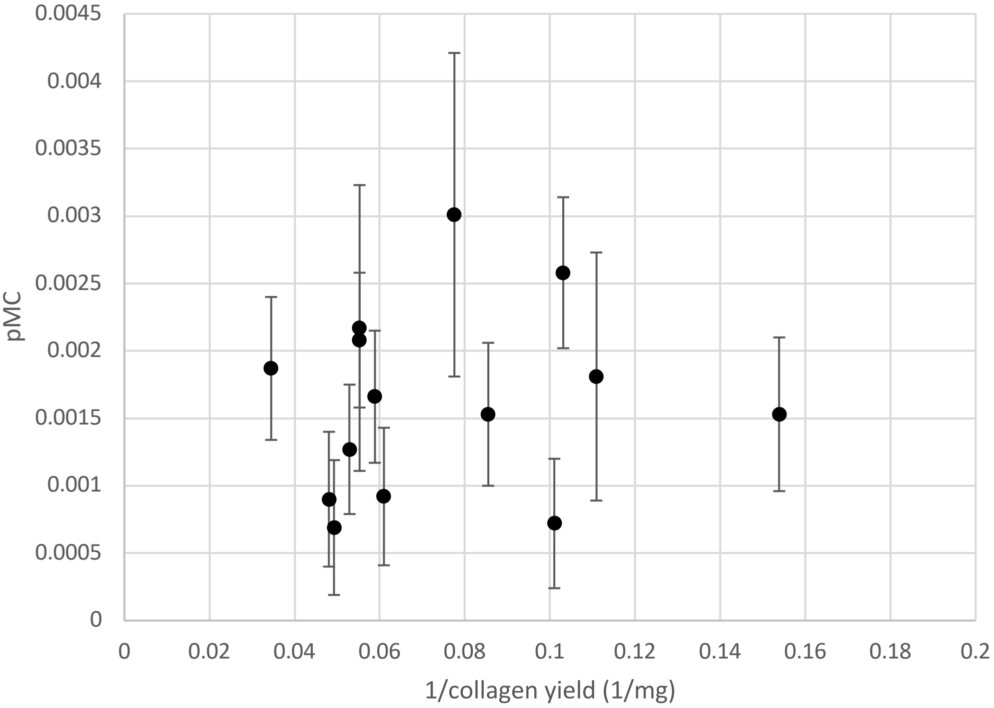

Figure S3.2 Chart showing pMC compared to yield for Latton Mammoth samples.

The amount of modern carbon contamination present in each sample due to the pretreatment process at UCL was calculated by multiplying the mg of carbon per sample, as measured by the IRMS during the burn process, by the pMC measured by the AMS. The error-weighted mean of these measurements is 0.0029 ± 0.0003 mg. The mass fraction of modern carbon for each sample is then calculated based on the burn weight of each sample (it comes to about 1% at normal burn weights), and this mass fraction then subtracted from the measured pMC for each sample. The corrected dates tend to be slightly older, due to the removal of contaminating modern carbon in the calculation. Table S3 below shows the uncorrected and corrected dates for samples discussed in the paper.

Table S3 Corrected and uncorrected radiocarbon dates.

Project sample code	ORAU date code	Uncorrected date	Corrected date code	Corrected date
UPN-221	OxA-V-2793-57	19030±80	OxA-V-2793-57C	19140±80
UPN-222	OxA-V-2797-20	19090±90	OxA-V-2797-20C	19200±90
UPN-223	OxA-V-2804-43	19000±80	OxA-V-2804-43C	19110±90
UPN-223	OxA-V-3002-12	18965±78	OxA-V-3002-12C	19130±80
UPN-231	OxA-V-2748-22	19180±90	OxA-V-2748-22C	19300±90

S4 Pretreatment methodologies used in the previously published radiocarbon bates (OxA-9737, OxA-9738, OxA-9739, ETH-34750)

OxA-9737, OxA-9738 and OxA-9739 were prepared and analysed at the Oxford Radiocarbon Accelerator Unit (ORAU), United Kingdom. The samples were prepared using ultrafiltration, and analysis was performed by accelerator mass spectrometry (AMS) with paired isotope ratio mass spectrometry (IRMS) analysis. The OxA-9737 sample was measured to have a carbon content of 27%, which would not meet present-day quality criteria for a bone collagen sample to be dated at ORAU. OxA-9738 was measured to have an atomic C/N ratio of 3.5, which would also not meet present-day quality criteria for a sample to be dated at ORAU. Further, all three samples were processed at ORAU using the original ultrafiltration method employed at the laboratory in 2000[24]. This was before it was recognised that the ultrafilters could be a source of additional sample contamination if appropriate cleaning procedures were not implemented[24]. While there is nothing to indicate that the OxA-9737, OxA-9738 and OxA-9739 samples were contaminated by the ultrafiltration procedure used at the time, we deemed it appropriate to re-date all three samples using present-day methods and quality control assessments.

ETH-34750 was prepared and analysed at the ETH Laboratory of Ion Beam Physics in Zurich, Switzerland. The sample was prepared using ultrafiltration. Analysis was performed by AMS, without paired IRMS analysis. The absence of IRMS analysis means that quality control parameters that are now routinely checked in the process of radiocarbon dating bone collagen samples (%C, atomic C/N ratio) could not be measured. As such, it was deemed appropriate to re-date this sample.
